# Supplementary material for: Diagnostic host gene signature for distinguishing enteric fever from other febrile diseases
Source: EMBO Mol Med. 2019 Aug 30;11(10):e10431. doi: 10.15252/emmm.201910431 (PMC6783646; doi:10.15252/emmm.201910431)
Supplement: Supplementary file 1 — Appendix [file EMMM-11-e10431-s001.pdf]

Diagnostic host gene signature for distinguishing enteric fever from other febrile diseases

Blohmke *et al.*

Appendix FIGURES and TABLES

Table of contents:

- **Appendix Figure S1:** Differentially expressed genes and BTMs of sEF cases from Nepal.
- **Appendix Figure S2:** Superset quality control.
- **Appendix Figure S3:** Signature identification using a re-designed discovery and validation cohort.
- **Appendix Figure S4:** Expression of the 7-genes signature identified during the multiclass classification analysis.
- **Appendix Figure S5:** Prediction of Oxford CHIM samples part of the unknown cohort.
- **Appendix Figure S6:** Correlation between microarray and qPCR gene expression.

- **Appendix Table S1:** Overlap of modular expression between enteric fever cohorts.
- **Appendix Table S2:** Overview of datasets used in this study.
- **Appendix Table S3:** Contingency tables for the two-class and multi-class prediction problems.
- **Appendix Table S4:** Prediction accuracies and misclassified samples (2-class problem).

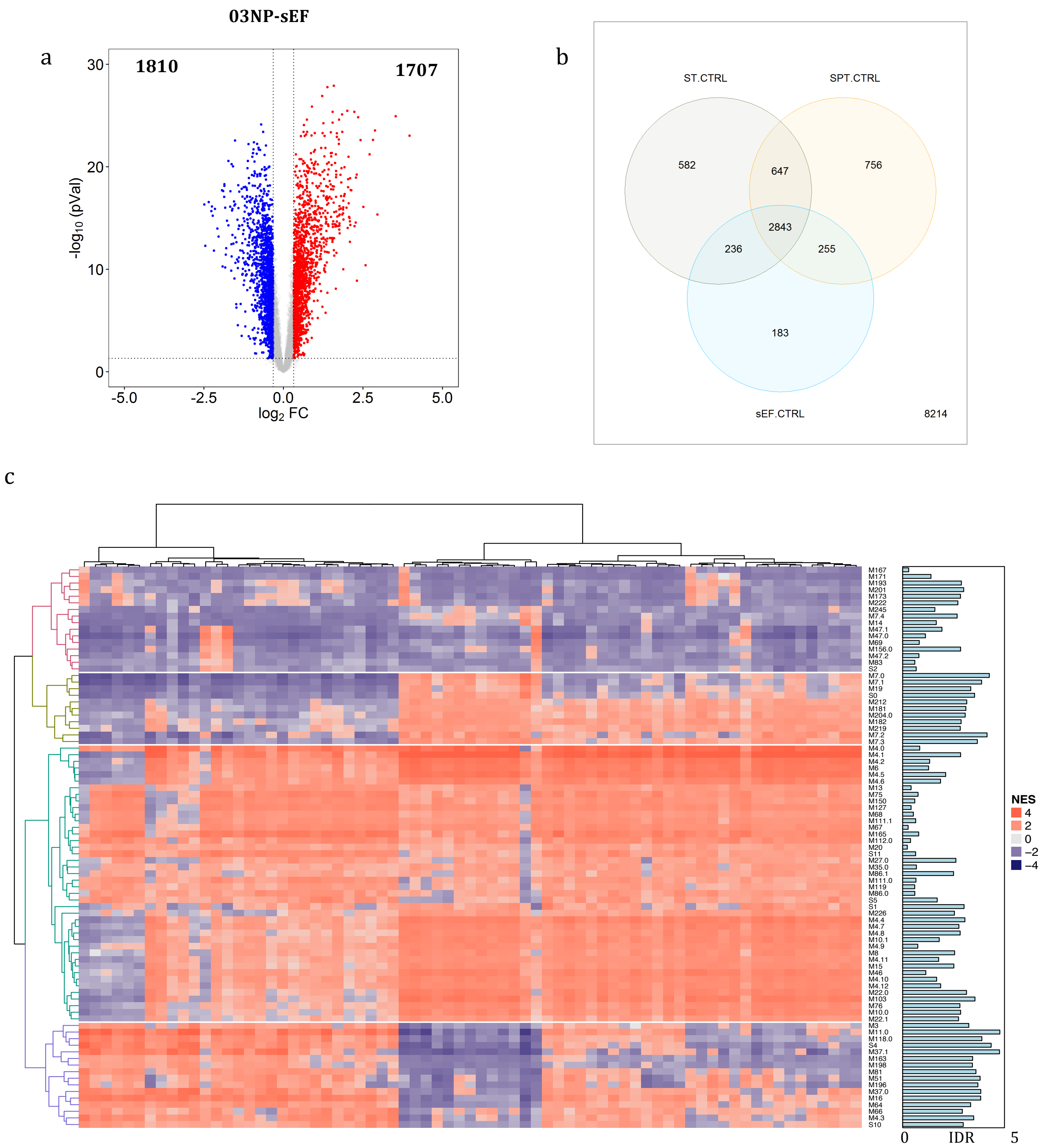

Appendix Figure S1: Differentially expressed genes and BTMs of sEF cases from Nepal. (**a**) Volcano plot of differentially expressed genes in the Nepali sEF cohort (blue: down-regulated; red: up-regulated compared to healthy community controls). (**b**) Venn diagram representing the overlap of DE genes in the Nepali ST, SPT and sEF cases. (**c**) ssGSEA heatmap of BTMs significantly expressed ($p<0.05$) in at least 60% of sEF samples. Bar plot panel represents the interdecile range (IDR) for each BTM across all sEF cases. NES: Normalized Enrichment Score.

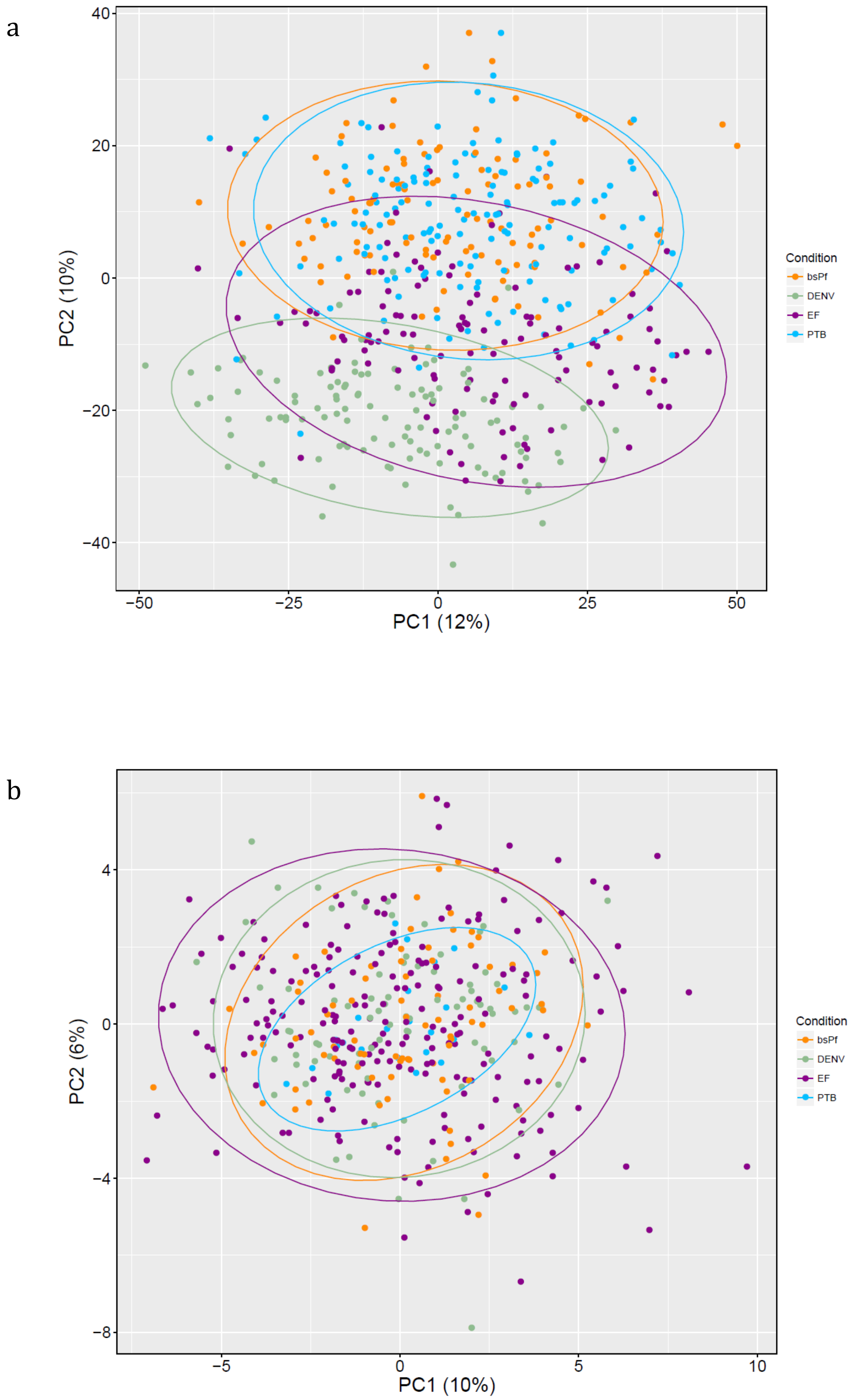

Appendix Figure S2: Superset quality control. (**a**) PCA plot based on the 500 most variable genes (IQR) of enteric fever cases (EF), malaria cases (bsPf), dengue cases (DENV) and TB cases (PTB) after batch correction. (**b**) PCA of all control samples for each disease cohort after batch correction.

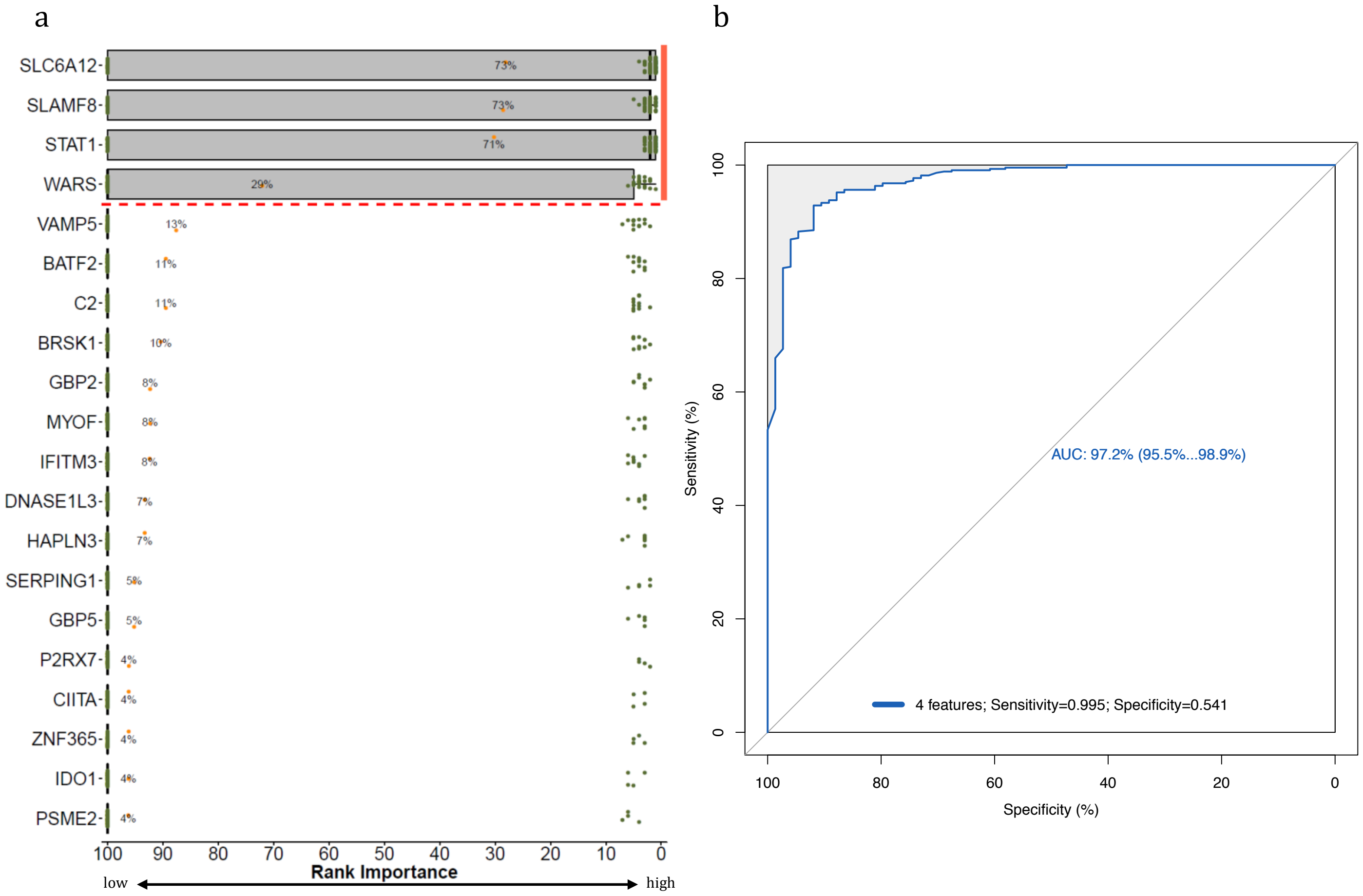

Appendix Figure S3: Signature identification using a re-designed discovery and validation cohort. (**a**) Ranking of genes by their selection frequency into the diagnostic signature out of 100 iterations (orange dot) during the 2-class classification. Y-axis = genes ranked by selection frequency. X-axis = importance measure of each gene across all 100 iterations. Green dots: Importance measure for each gene per iteration. A cut-off of 25% was chosen to detect a putative diagnostic signature consisting of 4 genes (orange bar). (**b**) Prediction of the validation cohort using the 4 genes identified in (a).

a

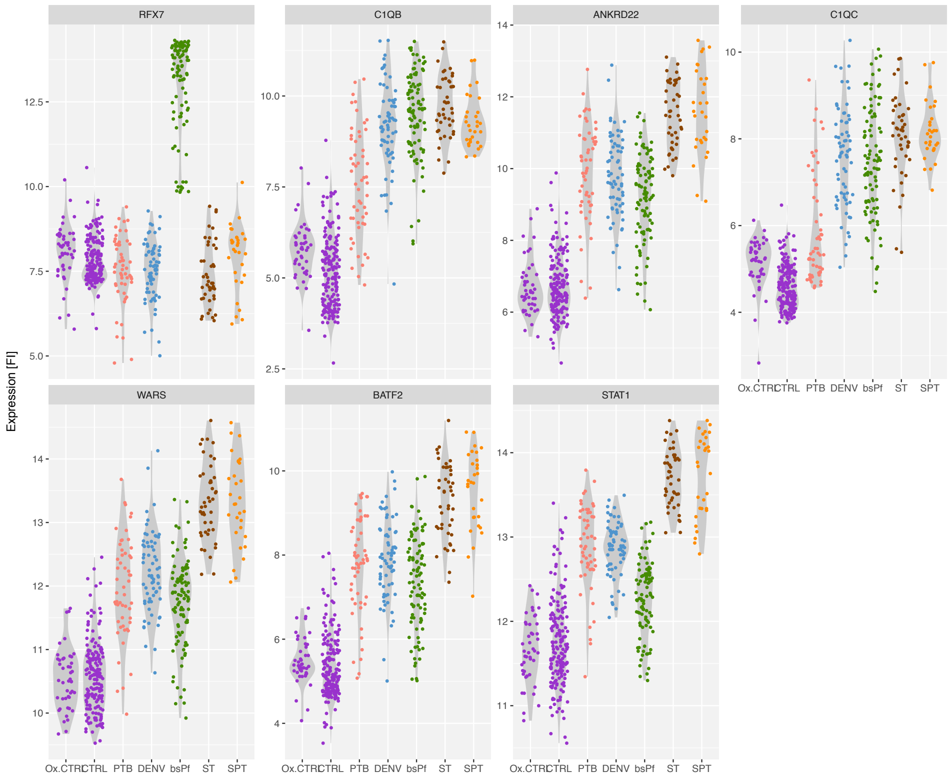

b

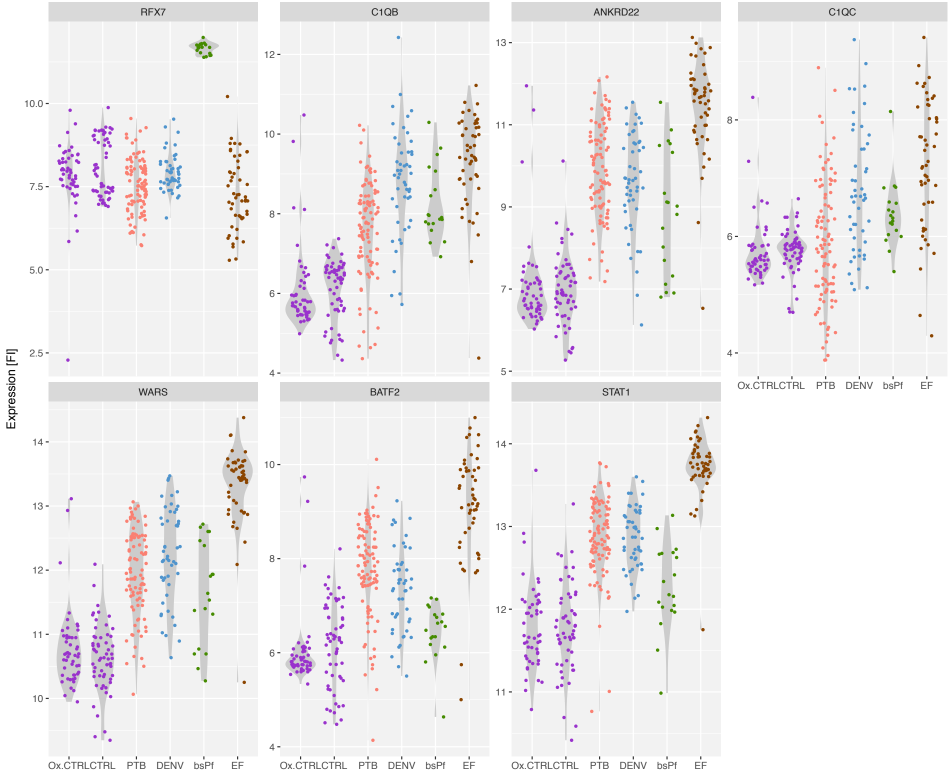

Appendix Figure S4: Expression of the 7 target genes identified during the multiclass classification analysis in each sample of the discovery (**a**) and validation cohort (**b**).

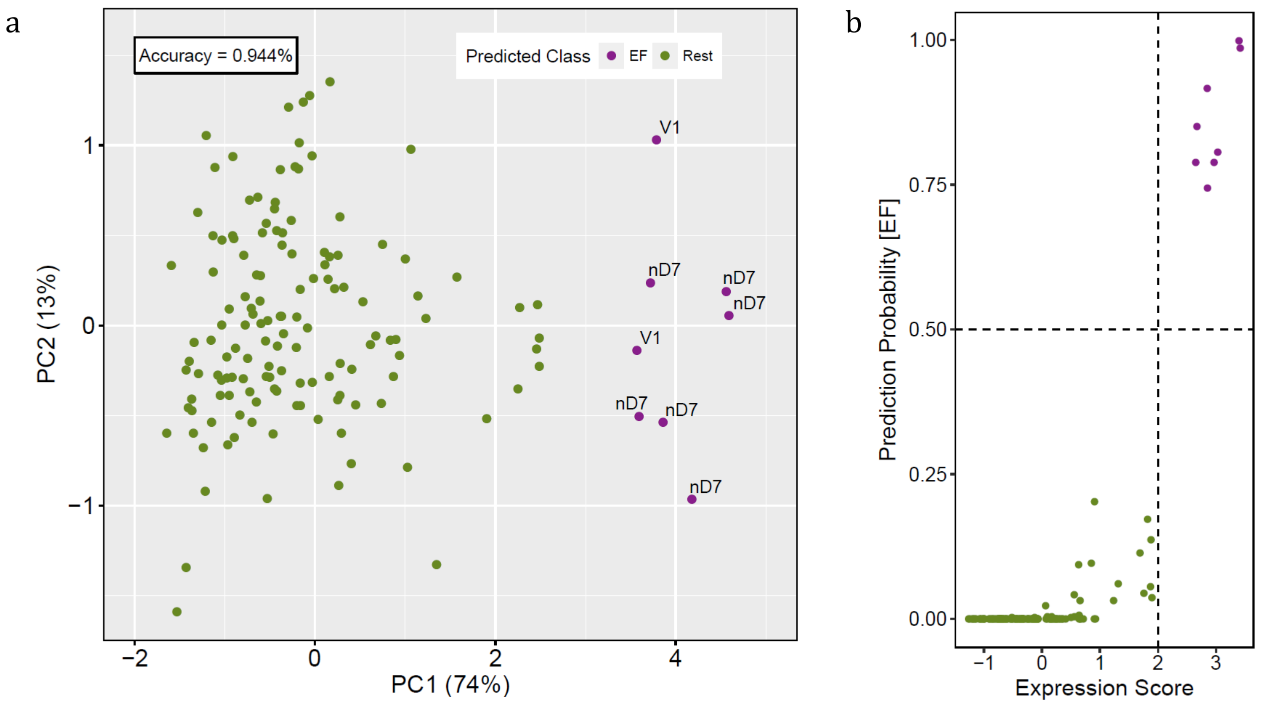

Appendix Figure S5: Prediction of Oxford CHIM samples part of the unknown cohort. (**a**) PCA of Oxford pre-challenge baseline samples and nD7 samples based on the expression values for the 5-gene diagnostic signature (2-class classification) coloured by predicted class membership (green: REST, purple: EF). (**b**) Dot plot of prediction probabilities against a combined expression score for each sample coloured by predicted class membership.

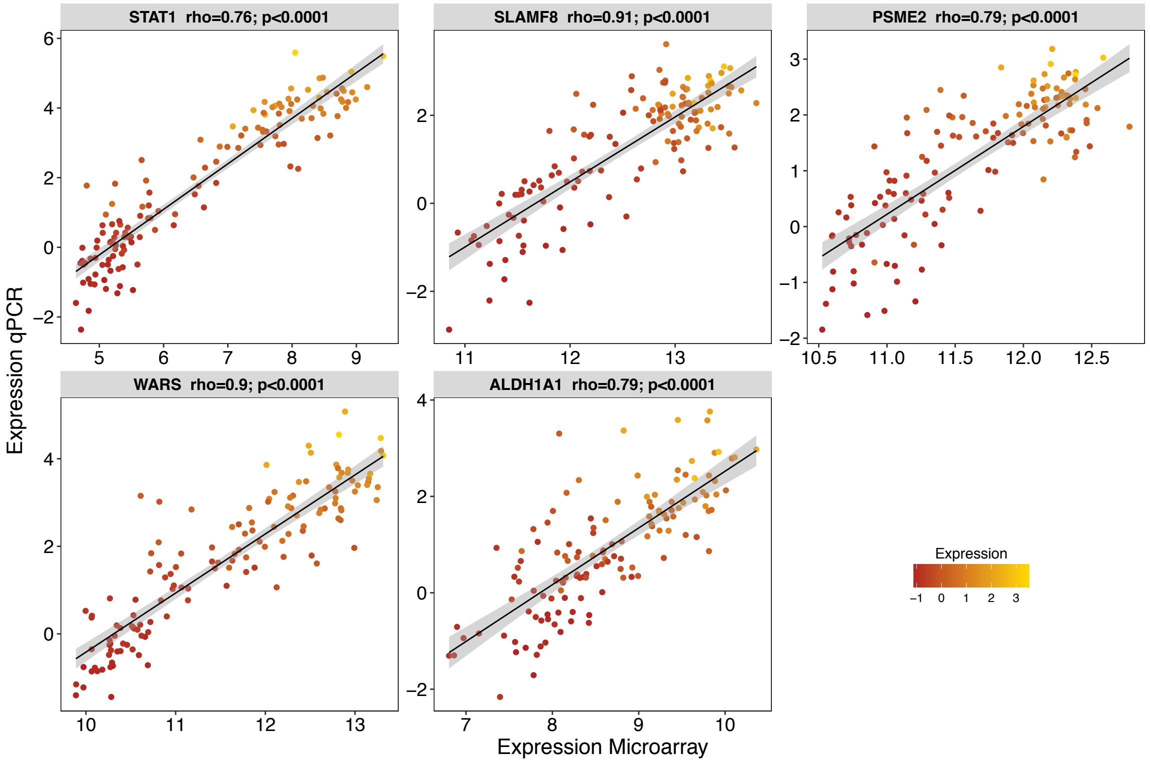

Appendix Figure S6: Spearman correlation of expression values of the 5-gene diagnostic signature derived from microarrays or qPCR.

	# enriched BTMs*	T1-ST	T1-nD7	03NP-SPT	03NP-ST	P1_SPT	P1-nD7
T1-ST	74	100	24	42	42	58	46
T1-nD7	64	28	100	34	34	28	33
03NP-SPT	54	57	41	100	91	69	33
03NP-ST	55	56	40	89	100	67	33
P1-SPT	65	66	28	57	57	100	37
P1-nD7	55	62	38	33	33	44	100

*Benjamini-Hochberg adjusted p<0.01

Appendix Table S1: Overlap of significantly enriched BTMs between different study groups (in percent).

Set	Study abbreviation	Study	Clinical and/or diagnostic criteria	Samples	Age	Platform	Other	Repository ID	Publication (PMID)
Discovery Cohort	***T1***	Oxford Typhoid 1	Temperature >38C, 12hrs and/or positive BC	Pre-challenge baseline (D0), acute typhoid fever (ST)	Adults	Illumina HT-12 V4.0	–	E-MTAB-3423	Blohmke et al.
	P1	Oxford Paratyphoid 1	Temperature >38C, 12hrs and/or positive BC	Pre-challenge baseline (D0), acute paratyphoid fever (SPT)	Adults	RNAseq	–	GSE113867	Current study
	03NP	Enteric Fever Nepal (03NP)	positive BC	healthy community controls (CTRL), acute enteric fever (ST; SPT)	Adults	Illumina HT-12 V4.0	–	GSE113867	Current study
	GSE34404	Malaria Benin	positive Parascreen, thick blood smear	acute patiente (bsPf), CTRL	Children (<10 years)	Illumina HT-12 V4.0	HIV+ excluded	GSE34404	Idaghdour et al.
	GSE19491	TB (UK, RSA)	culture positive respiratory sample	acute patients (PTB), CTRL	Adults	Illumina HT-12 V3.0	HIV+ excluded	GSE19491	Berry et al.
	GSE28991	DENV (Singapore 1)	<72hrs fever; qRT-PCR	acute patients (DENV), convalescent CTRL	Adults	Illumina HT-12 V4.0	–	GSE28991	N/A
	GSE25001	DENV (Vietnam)	<72hrs fever; NS-1 positive ELISA, qRT-PCR	acute patients (DSNV), convalescent CTRL	Adults	Illumina humanRef-8 v2.0	–	GSE25001	Hoang et al.
Validation Cohort	***T2***	Oxford Typhoid 2	Temperature >38C, 12hrs and/or positive BC	Pre-challenge baseline (D0), acute typhoid fever (ST)	Adults	Illumina HT-12 V4.0	–	GSE113867	Current study
	GSE51808	DENV (Thailand)	<72hrs fever; NS-1 positive ELISA, qRT-PCR	acute patients (DENV), HC	Adults	[HT_HG-U133_Plus_PM] Affymetrix	DENV1-3	GSE51808	Kwissa et al.
	GSE28405	DENV (Singapore 2)	undifferentiated fever; qRT-PCR	acute patients (DENV), convalescent CTRL	Adults	Sentrix HumanRef-8 Illumina	DENV1-3	GSE28405	Tolfvenstam et al.
	GSE64338	Malaria (Rwanda)	positive malaria rapid Dx test, thick blood smear	acute patients (bsPf), convalescent CTRL	Adults	[HuGene-1_0-st] Affymetrix	HIV+ excluded	GSE64338	Subramaniam et al.
	GSE37250	TB (South Africa)	culture confirmed	Active TB only (PTB)	Adults	Illumina HT-12 V4.0	HIV+ excluded	GSE37250	Kaforou et al.
	GSE37250	TB (Malawi)	culture confirmed	Active TB only (PTB)	Adults	Illumina HT-12 V4.0	HIV+ excluded	GSE37250	Kaforou et al.
Unknown Cohort	***03NP***	Enteric Fever Nepal (03NP)	Negative BC	febrile, culture-negative, suspected EF (sEF)	Adults	Illumina HT-12 V4.0	–	GSE113867	Current study
	T1	Oxford Typhoid 1	Negative BC	Pre-challenge baseline (D0), challenged but healthy day 7 sample (nD7)	Adults	Illumina HT-12 V4.0	–	E-MTAB-3423	Blohmke et al.
	P1	Oxford Paratyphoid 1	Negative BC	Pre-challenge baseline (D0), challenged but healthy day 7 sample (nD7)	Adults	RNAseq	–	GSE113867	Current Study
	T2	Oxford Typhoid 2	Negative BC	Pre-challenge baseline (D0), challenged but healthy day 7 sample (nD7)	Adults	Illumina HT-12 V4.0	–	GSE113867	Current Study
qPCR	***03NP***	Enteric Fever Nepal (03NP)	positive BC	healthy community controls (CTRL), acute enteric fever (ST; SPT); febrile, culture-negative, suspected EF (sEF).	Adults	Illumina HT-12 V4.0	–	GSE113867	Current study
	**Vi-TCV study**	Oxford Typhoid Challenge	Temperature >38C, 12hrs and/or positive BC	Pre-challenge baseline (D0), acute typhoid fever (TD)	Adults	qPCR	–	NA	Jin et al.

Appendix Table S2: Datasets included in the discovery, validation, unknown and qPCR cohort. Column "Samples" signifies the samples of each dataset used in each part of the analysis (Discovery, Validation, Unknown cohort). D0 = Healthy, pre-challenge baseline samples from the challenge studies; CTRL = healthy community or convalescent controls; ST = *S.* Typhi; SPT = *S.* Paratyphi A; DENV = dengue virus samples; bsPf = blood-stage *Plasmodium falciparum* samples; PTB = pulmonary Tb samples; sEF = febrile, culture-negative, suspected enteric fever samples; nD7 = day 7 samples derived from participants of the challenge study who stayed well the entire 14 day challenge period. Note: some studies repurposed from GEO include several datasets. For example, GSE37250 by Kaforou *et al.* contains two cohorts of tuberculosis patients – one cohort from South Africa and one cohort from Malawi.

(a) Contigency table of class prediction in the 2-classification

		Reference	
		Rest	EF
Pred.	Rest	266	6
	EF	8	44

Sensitivity	0.97
Specificity	0.88

(b) Contigency table of class prediction in the multiclass classification

		Reference				
		CTRL	Malaria	DENV	EF	PTB
Prediction	CTRL	105	0	1	2	15
	Malaria	0	19	3	0	1
	DENV	2	0	31	0	23
	EF	1	0	5	46	13
	PTB	1	0	9	2	45

	CTRL	Malaria	DENV	EF	PTB
Sensitivity	0.963	1	0.632	0.92	0.463
Specificity	0.916	0.986	0.909	0.93	0.947

Appendix Table S3. (**a**) Contingency table of class membership following the 2-class classification. (**b**) Contingency table of class membership following the multiclass classification.

2-Class Classification: Unknown Cohort prediction accuracies

2-Class Classification Oxford samples			
Algorithm	EF	Rest	Accuracy
RF	8	136	0.944

Misclassified Oxford Samples (Unknowns)			
Sample	Predicted	Time point	Study
P1_73_D7	EF	nD7	P1 (OXF)
P1_77_D0	EF	V1	P1 (OXF)
T1_27_nV1	EF	V1	T1 (OXF)
T2_1831	EF	nD7	T2 (OXF)
T2_3691	EF	nD7	T2 (OXF)
T2_6297	EF	nD7	T2 (OXF)
T2_8628	EF	nD7	T2 (OXF)
T2_9631	EF	nD7	T2 (OXF)

Appendix Table S4: Prediction accuracy and overview of misclassified samples following prediction using the 5-gene 2-class signature of the Oxford samples included in the unknown cohort.
